# Principal component and cluster analyses of production and fertility traits in Red Sindhi dairy cattle breed in Brazil

**DOI:** 10.1007/s11250-019-02009-7

**Published:** 2019-08-01

**Authors:** Raquel Rodrigues Costa Mello, Letícia Del-Penho Sinedino, Joaquim Esquerdo Ferreira, Sabrina Luzia Gregio de Sousa, Marco Roberto Bourg de Mello

**Affiliations:** 1 Department of Animal Evaluation and Reproduction, Federal Rural University of Rio de Janeiro (UFRRJ), BR 465, Km 07, Seropedica, Rio de Janeiro Brazil; 2 Department of Animal Sciences, University of Florida (UF), 2250 Shealy Drive, Gainesville, FL 32911 USA; 3 Department of Animal Production, Federal Rural University of Rio de Janeiro (UFRRJ), BR 465, Km 07, Seropedica, Rio de Janeiro Brazil

**Keywords:** Multivariate analysis, Milk yield, Reproductive efficiency

## Abstract

The objective of this study was to investigate the relationship among functional traits (age at first calving (AFC), calving interval (CI), reproductive efficiency (RE), total milk yield (TMY), and lactation period (LP)) in Red Sindhi breed through multivariate techniques. For this goal, performance data provided by the Brazilian Association of Zebu Breeders related to 560 Red Sindhi dairy cattle from 28 different herds in Brazil, born in the period from 1987 to 2011, were used. Principal component analysis with correlation matrix was used to find the relationship among AFC, CI, RE, TMY, and LP. It was found that for all functional traits, first 3 principal components explained more than 90% of the total variation. Clustering analysis was performed based on Tocher method, and results showed physiological relationships among functional traits. By cluster analysis, twelve different groups were generated from the pool of Sindhi herds analyzed, with a great homogeneity among females for the traits evaluated and only few females generating separate groups. Four hundred and twenty-nine females were clustered in one group, representing 76.60% of the genotypes. Total milk yield (TMY) showed 71.92% of the total variation, and age at first calving (AFC) contributed with 23.06% of the variation, being the two most important traits for the variability of the data set. In conclusion, the multivariate procedures were effective in generating the correlations among the functional traits, showing that CI is correlated with RE and all these functional traits are related with total milk yield.

**Electronic supplementary material:**

The online version of this article (10.1007/s11250-019-02009-7) contains supplementary material, which is available to authorized users.

## Introduction

Multivariate techniques, such as cluster and principal component analyses, could be used to find the loadings or factors that explain the highest variation in the data set over dependent variables, providing a tool to build relationships that allow the clustering of animals by similar productive traits and correlations between different characteristics evaluated. Once identified, groups of animals with desirable traits can be selected for animal breeding programs in order to enhance productivity and fertility (Karacaören and Kadarmideen 2008; Buzanskas et al. 2013; Jolliffe and Cadima 2016; Lopes et al. 2016). In addition, multivariate analyses are useful for addressing relevant decisions that reach male and female offspring with higher average performance related to previous generations by heterosis, increasing the variability of the population (Gianola and Sorensen 2004; Lopes et al. 2013; Moraes et al. 2015; Fraga et al. 2016). Moreover, multivariate statistical techniques might reveal relationships that would not be possible with univariate statistical techniques (Usai et al. 2006; Bolormaa et al. 2010).

Thus, it is essential to know genetic associations between the economic importance traits in the reason that they are correlated in magnitude and direction (Sonesson and Meuwissen 2000; Savegnago et al. 2011; Porto-Neto et al. 2013; Osorio-Avalos et al. 2015). Thus, multivariate statistical techniques might generate not possible interpretations if univariate statistics was used. According to Gressler et al. (2000) and Cardoso et al. (2003), multivariate analyses are useful for addressing relevant decisions in animal breeding programs that reach to obtain male and female animals with higher average performance with regard to previous generations by heterosis, increasing the variability of the population.

Red Sindhi is a zebu (*Bos primigenius indicus*) breed named after its region of origin, which is the north part of the Sindh province of Pakistan. Climate in that region is very hot and very dry, with maximum temperatures frequently rising above 46 °C and average rainfall around 150 to 180 mm per year. Purebred herds of this breed can also be found in India and many other countries that imported animals from Pakistan. In this way, Brazil also imported Red Sindhi animals, whereas those animals have been demonstrating good adaptation to a variety of environmental conditions (Faria et al. 2004; Khatri et al. 2004; Pundir et al. 2007; Mello et al. 2016; Panetto et al. 2017a; Carvalho et al. 2018).

In the year 1930, some red zebu animals were selected among many other cattle being imported at that moment. They were kept breeding in the State of São Paulo, for about two decades without any breed classification. Later, those animals were identified as belonging to the Red Sindhi breed. In a second moment, there was an official importation of 28 females and 3 males selected from purebred Red Sindhi herds in Pakistan, which arrived at the Brazilian island of Fernando de Noronha in the year 1952, whereas the establishment of the Brazilian Red Sindhi herds was based on these two small founder populations only (Leite et al. 2001; Moura et al. 2009; Mello et al. 2013; ABCZ 2014; Silva et al. 2014).

Today, most herds of Red Sindhi cows are located in the Northeast and Southeast regions of Brazil. This breed is generally considered suitable to be used in dual-purpose production systems, dairy and beef. However, selection in the Southeast herds was more emphasized on beef traits, in contrast to the Northeast herds that selected mostly for dairy traits. In the Northeast of Brazil, there is a predominance of harsh environmental conditions, with high average temperatures and very low precipitation. The Red Sindhi has been well adapted to those conditions, and most breeders claim that cows can keep good body condition scores, good fertility, ability for milk production, and optimal feed efficiency, even in these very harsh environments (Berman 2011; Mello et al. 2014; Cardoso et al. 2015; Saraiva et al. 2015; Oliveira et al. 2017; Panetto et al. 2017b). According to ABCZ (2014), Red Sindhi dairy cattle have shown averages of 530 days for calving interval, 245 days for service period, 75% for reproductive efficiency, 7 kg per day for milk yield, 265 days for lactation period, and 1875 kg for total milk yield. However, there are no studies using multivariate techniques to address productive and reproductive efficiencies in Red Sindhi dairy cows. Therefore, this type of analysis is important to develop new strategies aiming at improving productive and reproductive performances in Red Sindhi dairy cattle breed in Brazil.

The aim of this study was to investigate the relationship among functional traits (age at first calving (AFC), calving interval (CI), reproductive efficiency (RE), total milk yield (TMY), and lactation period (LP)) in the Red Sindhi breed through multivariate techniques, in order to give directions in Red Sindhi breeding programs.

## Material and methods

Functional traits, based on the averages from each animal, such as AFC (in days), CI (in days), RE (in percentage), TMY (in kilograms), and LP (in days), were analyzed. Information in regard to milk yield and lactation period, being the main trait yield up to 305 days, was reported. Similarly, information about age at first calving, calving interval, and reproductive efficiency was analyzed. For AFC, CI, and RE, dates at first and last calving were considered, using calving order at 1st to 17th.

In regard to age at first calving, this one was calculated by subtracting the birth date for the first calving date. Related to calving interval, those ones up to 16 intervals were considered. For reproductive efficiency, the formula RE = calving number × (365 × 100) calving mean interval was adapted, being calving interval specific in days, according to Guimarães et al. (2002).

In this sense, data related above regarding reproductive efficiency and milk yield from 560 Red Sindhi dairy cows properly registered at Brazilian Association of Zebu Breeders (ABCZ), located in Uberaba, MG, Brazil, born from 1987 to 2001, were analyzed. This study was carried out from the Brazilian Association of Zebu Breeders database collection, whereas the data were recorded in files of Microsoft Excel 2010® by the Superintendência de Melhoramento Genético, from ABCZ.

All animals enrolled in this study are Red Sindhi dairy cows included in Red Sindhi breeding program and participants of the breed official milk control referring to 28 herds in Paraiba, Rio Grande do Norte, Minas Gerais, Ceara, Distrito Federal, São Paulo, Espirito Santo, and Para states. For each herd and Brazilian location, nutritional and reproductive management was specific. For this reason, measuring the eminence of forage and pluviometry seasonality was not possible.

Data were subjected to multivariate statistical procedures of principal component and clustering analyses, as well as performed by GENES® (Cruz 2006) software. The software GENES is compatible with IBM PCs and requires the Windows operating system. Some configuration settings are indispensable, such as a screen resolution of 1024 × 768 (large fonts) and the use of a decimal symbol expressed by points. The package comprises 257 executable projects and 131 text documents in rtf format, occupying about 285Mbytes, available in English, Spanish, and Portuguese.

The use of GENES® to designate multivariate analysis represents a large number of methods and techniques that simultaneously use all variables in the analysis, interpretation, and processing of the data set from a biological phenomenon under study. The mathematical complexity, typical of multivariate methods, has inhibited the transfer of the underlying stochastic fundamentals and principles to the researchers. However, the key part, which is the statistical inference, has been stimulated through the use of well-constructed software with a user-friendly interface for researchers.

In the program GENES®, the scientist will find the analysis of structural simplification, such as principal component and canonical variable analyses; association analysis, such as path analysis, canonical correlations, and factor analysis; analysis of diversity, such as discriminant analysis (based on principal components); measures of dissimilarity, based on continuous, multicategoric, or binary phenotypic quantitative variables; analysis of molecular data from dominant or codominant markers; cluster analysis, such as Tocher optimization method, hierarchical, graphic dispersion, and 2D and 3D projection; and identification of more and less similar accessions.

One the major contributions of computing is that phenomena can be studied by simulating a complex situation in which parameters and constraints are established, so that the effect of certain controllable factors can be conveniently studied. Simulation is defined as a way of imitating the behavior of a real system by computational resources to study its functioning under alternative conditions, involving certain types of logic models to describe, as best as possible, the natural system. Simulations are highly useful in genetic studies in various contexts, including studies of populations, of the individual, or of the proper genome. They require the development of appropriate biological models to represent the phenomena of interest as ideally as possible by researchers and suitable procedures of processing by programmers, according to the parameters and constraints, so that the influence of certain factors can be assessed.

A scatter plot from scores for better view of each sample element within the data set was created. Cluster analysis implied on two stages, the first one, the estimation of dissimilarity measures, and the second one, the adoption of a cluster method. For dissimilarity measure, the mean Euclidean distance was chosen. Due to the fact that the Euclidean distance is influenced by measurement range and number of the variables, as well as the correlation among them, variables for normal distribution with average 0 and variable 1 were standardized.

Furthermore, the Euclidean distance increases as the number of variables is added (Cruz 2006). Thus, for overcoming this problem, the mean Euclidean distance was employed, wherein the value of the Euclidean distance was divided by the square root of the variable number. The Tocher (non-hierarchical) cluster method was employed in the reason of the individual’s large number might hinder the recognition of homogeneous groups by simple visual examination of graphics created by hierarchical methods.

Thus, the Tocher method started from the dissimilarity matrix, identifying the most similar pair of individuals and these ones constituting the initial group. Following, the possibility of new individual mean distance within the group which was smaller than the ones among any groups was evaluated. As an increase in limit on the intra-group distance average, including or not new element in the group, the dissimilarity measure maximum value reported by smaller distances set was adapted as criterion (Cruz 2006).

## Results

The main objective of this study was to conduct a phenotypic analysis exploring relationships and dependencies among a group of traits that significantly affect the function of dairy cows (functional traits). This investigation was based on data collected from Red Sindhi herds kept in several farms around regions of Brazil, using principal component and clustering analyses. These statistical methods and concepts such as principal component and clustering analyses are equally applicable to large volumes of data collected by the Brazilian Association of Zebu Breeders (ABCZ) with regard to the Red Sindhi breed. Although limited in data size, some of the analyses were statistically significant and will be useful.

**Fig. 1:**
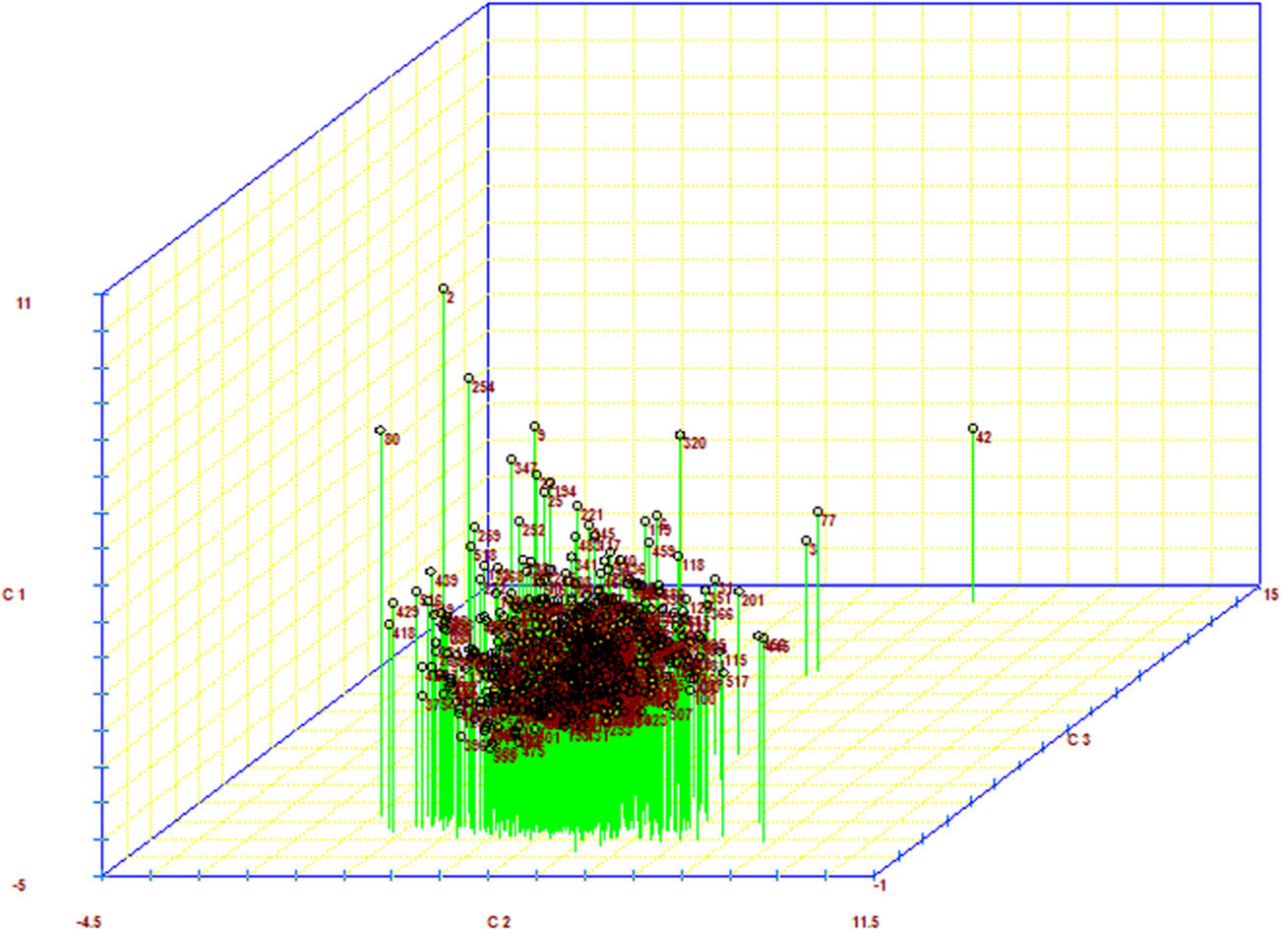
Scatter plot contrasting the principal components 1 (PC1), 2 (PC2), and 3 (PC3)

For this purpose, Table 1 shows the matrix of correlation among the standard variables used in the analysis of main components. Table 2 shows the principal components, eigenvalues, percentage of variance explained by the components, and accumulated percentage of variance explained by the components. Table 3 shows the correlations between the variables and the main components estimated. Supplementary Table shows Sindhi female groups established by the Tocher method based on the dissimilarity expressed by Euclidean distance standardized mean. Table 4 shows the averages of the traits for the twelve groups formed by the Tocher method. Table 5 shows the relative contribution of traits for the divergence among the 560 female Sindhi. Figure 1 shows the scatter plot contrasting the components 1 (PC1), 2 (PC2), and 3 (PC3).

**Table 1:** Matrix of correlation among the standard variables used in the principal component analysis

Variables	AFC	CI	RE	TMY	LP
AFC	1.0000	− 0.0335	0.0071	0.0005	0.0969
CI	− 0.0335	1.0000	− 0.8417	0.0402	0.0165
RE	0.0071	− 0.8417	1.0000	− 0.0450	− 0.0041
TMY	0.0005	0.0402	− 0.0450	1.0000	0.6903
LP	0.0969	0.0165	− 0.0041	0.6903	1.0000

*AFC*, age at first calving; *CI*, calving interval; *RE*, reproductive efficiency; *TMY*, total milk yield; *LP*, lactation period

**Table 2:** Principal components, eigenvalues, percentage of variance explained by the components, and accumulated percentage of variance explained by the components

Principal components	Eigenvalues	%VPC	%VPC (accumulated)
PC1	1.8590	37.18	37.18
PC2	1.6819	33.63	70.81
PC3	0.9986	19.97	90.79
PC4	0.3032	6.06	96.85
PC5	0.1572	3.14	100.00

*PC*, principal components; *eigenvalues*, variances; *%VPC*, percentage of variance explained by the components

**Table 3:** Correlations among the variables and the principal components (PC) estimated

Correlations	CP1	CP2	CP3	CP4	CP5
IPP	− 0.0135	0.1420	0.9883	− 0.0522	− 0.0111
IDP	0.9214	− 0.2673	0.0158	0.0353	− 0.2794
ER	− 0.9201	0.2693	− 0.0461	0.0205	− 0.2798
PTL	0.3045	0.8598	− 0.1394	− 0.3851	− 0.0184
DL	0.2653	0.8824	− 0.0043	0.3880	0.0204

*AFC*, age at first calving; *CI*, calving interval; *RE*, reproductive efficiency; *TMY*, total milk yield; *LP*, lactation period

**Table 4:** Means of the variables regarding the 12 groups formed by the Tocher method

Groups	AFC (days)	CI (days)	RE (%)	TMY (kg)	LP (days)
1	1242.90	523.77	71.75	1439.23	214.84
2	1326.09	651.49	59.46	2809.75	319.03
3	1205.82	893.76	42.13	908.06	131.87
4	1198.40	409.66	89.40	576.80	76.70
5	1267.75	1556.68	24.24	2315.70	284.99
6	1451.38	404.40	90.52	2266.89	326.10
7	1265.00	478.00	76.39	6016.55	324.50
8	5375.50	489.00	74.95	1390.05	230.00
9	2122.50	427.57	85.51	3009.25	177.00
10	7370.00	539.38	67.67	1495.20	356.00
11	1119.00	2008.76	18.17	693.60	68.00
12	817.00	3919.50	12.09	2125.20	276.00
Minimum	405.00	366.00	12.08	45.60	15.00
Maximum	7370.00	3019.50	99.72	6329.60	365.00

*AFC*, age at first calving; *CI*, calving interval; *RE*, reproductive efficiency; *TMY*, total milk yield; *LP*, lactation period

**Table 5:** Relative contribution of traits for the divergence among the 560 Red Sindhi dairy cattle by the method of Singh (1981)

Variables	Variance (**S**_**.j**_)	Value (%)
TMY	248296894892.4695	71.9206
AFC	79618758911.0	23.0620
CI	15105771881.8236	4.3755
LP	2139860196.6864	0.6198
RE	7639396.6991	0.0221

*TMY*, total milk yield; *AFC*, age at first calving; *CI*, calving interval; *LP*, lactation period; *RE*, reprodutive efficiency

## Discussion

Principal component analysis was performed using the explanatory variables based on a model. According to Morrison (1976), because the main components and the Euclidean distances are influenced by the scale of the variables, it is recommended to use standard variables with variance equal to unity. This standardization was performed by the GENES® software itself, which performs all the functions necessary for the application of the analyses.

Through the matrix of correlation between the variables, as shown in Table 1, it was possible to observe some traits that presented significant simple linear correlation with others, being therefore less important for the total variation. Thus, the variable reproductive efficiency (ER) was the least important to explain this variability, since it is a linear combination of the calving interval (CI), with a significant correlation with this variable (− 0.8417). The variable total milk yield (TMY) presented a high correlation with lactation period (LP), being 0.6903, indicating that both do not necessarily need to be maintained in future studies. On the other hand, the same variable TMY presented a non-significant correlation with CI, suggesting the maintenance of both characteristics in future studies with the Red Sindhi cows, since they are equally important for the evaluation of milk production systems.

Although the correlation between RE and CI was significant, it was negative in nature. In fact, the longer the calving interval, the lower the number of calves produced over the productive lives of cows, and therefore, the lower their reproductive efficiency. The correlation of negative nature observed between TMY and ER (− 0.0450) may be related to the antagonism between both variables (Table 1) (Chagas et al. 2007; Leroy et al. 2008; Barbat et al. 2010; Grimard et al. 2013).

Five principal components, as shown in Table 2, demonstrating that this technique was effective for maintaining as much information in terms of total variation from initial data, were obtained. The first component explained 37.18%, the second 33.63%, and the third 19.97% of the total variation. Investigation of these results shows that most of the variations are explained by the first 3 principal components for all traits, whereas more than 90% (90.79) of the total variations were explained by them.

According to Table 2, the variables were reduced in three components, of which two presented eigenvalue less than 0.7, being discarded according to the criteria proposed by Jolliffe (1972, 1973), who report that when the principal component analysis uses the correlation matrix, the number of discarded variables should be less than or equal to the number of components whose variance is less than 0.7. Thus, it is possible to discard the variables that few contribute to the discrimination of the data. The same model was used by Barbosa et al. (2006) and Santos et al. (2010) for the discard of variables. However, the purpose of our work was not to evaluate towards the discarding of variables, since it was not a large number of variables. Thus, this analysis allowed the discrimination of the most important and least important traits for the total variability, with more or less relevance.

The relative importance of the variables might be assessed by the magnitude of these variables’ correlation in regard to the first and last components presenting higher or lower relevance. Reproductive efficiency (− 0.2798) and lactation period (0.3880) presented the highest correlations, from the last principal component towards the first one, as shown in Table 3. However, the trait lactation period was highly correlated with the second principal component (0.8824), which accounted for 33.63% of total variation.

The main components estimated have identified the importance of the traits regarding the percentage of explanation for the total variance of the data set. In this sense, the traits considered most relevant to the variability of overall data, in the order of most important to less important, were CI, LP, AFC, TMY, and RE, and the last two traits were less important to the total variation, because they are highly correlated with the PC4 and PC5 components (Table 3). This fact is due to the traits highly correlated to the principal components with less variance showing practically insignificant variation (Mardia et al. 1997).

Thus, the three first principal components (PC1, PC2, and PC3) were used to contrast the females with larger or lower average performance for the traits in which they were correlated. Therefore, the variables CI, LP, and AFC were associated with PC1, PC2, and PC3, respectively. Moreover, the variables CI and RE were more associated with PC1, indicating that it showed greater genetic interdependence among themselves, while TMY and LP were more associated with CP2 and AFC to PC3 (Table 3). These results indicated that genetic variability in the traits associated with the PC1 or PC2 may cause genetic variability in the same direction in the others, except for AFC, which was more associated to PC3. Therefore, AFC had lower genetic association with the remaining variables. Thus, females can be selected by numerical scores generated by PC1 and PC2 to improve CI, RE, TMY, and LP, being the scores obtained by PC3 to improve the AFC (Table 3).

Santos et al. (2010) used principal component analysis to evaluate the formation of productive groups in Girolando breed females of three genetic groups (1/2, 3/4, and 7/8 Holstein × Gyr) with the purpose to discriminate the traits most important for milk yield in that breed. These authors obtained three components, which accounted for 82.84% of the total variation, being correlated with the three traits not subject to disposal in that study, genetic group (PC1), milk weight in the third milking (PC2), and age of the cow at the time of milk control (PC3). Thus, the analysis of the principal components, as well as being important for the discrimination of the most important traits, may also indicate those less relevant and therefore likely to be disposed in breeding programs when it has a higher number of variables and needs to eliminate the most redundant in order to have a better view of the groups by the retained components.

A scatter plot from principal component analysis is presented in Fig. 1. By the scores from principal components 1 (PC1), 2 (PC2), and 3 (PC3), the formation and differentiation of the female’s groups following the data standardization might be observed by the three-dimensional design. Great heterogeneity among cows with regard to principal components evaluated, with few cows clustering different and mutually exclusive groups, was observed. Females with genotypes 2, 42, and 80 were the most divergent, ones distancing from the average with regard to the others, and consequently graphically farther located. As a matter of fact, these cows individually formed a group. It was also observed that high similarity among cows prevented to view those more like productively, confirming the efficiency of the principal component analysis in which the three first components (PC1, PC2, and PC3) were sufficient to represent the segregation of the cows.

Clustering analysis by the Tocher method for forming productively homogeneous groups was employed. The cluster of the cows by this method, based on Euclidean distance, allowed the establishment of twelve groups, which relieve the view of the divergence between the cows, being them with the same pattern of similarity (Supplementary Table). Females 2, 42, and 80 formed distinct and mutually exclusive groups. The Tocher method enabled the cluster of 429 females (76.6% of the genotypes) on the first group, showing a strong homogeneity within this group. This indicates that although there are genotypes with great genetic divergence among them, more than half is similar for the traits evaluated, evidencing a narrow degree of relation among them.

This result might be expected; despite the fact that cows were from 28 different herds, there are only two Red Sindhi breeding centers in Brazil (Northeast and Southeast regions), being responsible by dispersion of most the cows to other states and regions. Thus, farmers might be employed the same dairy cows considered the best ones in their herds, contributing for the development of one cluster with a lot of productively homogeneous cows regarding the traits evaluated. Therefore, the clusters represent valuable information in choosing cows within the breeding programs, because the new populations to be established should be based on the magnitude of their genetic distances and the potential of these females.

In breeding programs that reach the generation of the best cows, considerable genetic divergence among them, as well as animals with desirable averages for the traits to be improved, is required. Thus, the highest and lowest Euclidean distances were estimated among the cows based on the traits (AFC, CI, RE, TMY, and LP). Estimates pointed the degrees of similarity and dissimilarity among the cows evaluated, and the results showed that the highest dissimilarity occurred between females 2 and 196 (Euclidean distance of 0.6800) and the highest similarity between females 192 and 219 (Euclidean distance of 0.0040).

In fact, cows 2 and 196 were from different states in Brazil (Paraiba and Minas Gerais states), with parents from different locations (Paraiba and São Paulo states), showing averages for the traits so divergent, which contributed to the greater distance between them, being them from different groups. Because they are in different locations, they may have been submitted to different management conditions. In contrast, cows 192 and 219 were from the same state in Brazil (Minas Gerais state), with parents from the same location (São Paulo state), showing averages for the traits so similar, which contributed to the greater proximity between them, being them from the same group. This may be due to the fact that these cows may have been submitted to the same type of management conditions.

According to Table 4, the twelve different groups presented particular averages for each one of the traits evaluated, which can target the selective breeding in each cluster according to the quantity or percentage of ideal females. Thus, groups 2 (63 females), 5 (8 females), 6 (13 females), 7 (2 females), 9 (2 females), and 12 (1 females) that represent 50.00% of the clusters showed averages for TMY above 2000 kg, with emphasis on groups 7 and 9. These two groups were formed by only two cows each, which probably stood out in milk yield because they came from the Northeast region (Ceara and Paraiba states), whereas they have been submitted to a management to improve genetically their milk production. In the same way, among the groups with the best averages for AFC, groups 11 and 12 stood out, being formed by only a cow each (genotypes 80 and 2), whereas the cow from genotype 2 had AFC of approximately 27 months. These two cows were from the Northeast region (Paraiba state), which has also targeted the Red Sindhi breed for sexual precocity.

It was also observed that a greater RE does not necessarily imply in a higher TMY, such as was confirmed by principal component analysis (− 0.0450) and observed by the averages of groups 4, 5, 10, and 12. Group 4, for example, showed RE greater than group 5, but since the TMY in that group was lower than that in group 5, the same was observed between groups 10 and 12 (Table 4). This fact can be related to the different averages for CI, whereas RE is directly related to it, and the lower the CI, the greater the number of offspring, and therefore, the greater will be the RE, as observed by groups 4, 6, 7, 8, and 9. Group 12 showed the lowest average for ER, showing higher CI (3919 days), while group 6 showed the highest average for ER, showing lower CI (404 days) (Table 4).

Regarding LP, the recommended period for dairy cattle is approximately 10 months, in order to have a birth for a year and higher milk yield per cow during her productive life. Thus, it becomes necessary to consider the LP as a variable to be studied in genetic and environmental factors, despite its high correlation with milk yield, in order to be able to use in the breeding programs for dairy cows, especially in countries with hot climate (Nobre et al. 1984; Milagres et al. 1988; Vasconcelos et al. 1989; Barbosa et al. 1994; Guimarães et al. 2002). In the present study, LP showed to be strongly correlated with TMY (0.6903). In fact, the groups with lower averages for total milk yield also had lower averages for lactation period, as shown in Table 4. Groups with lactation period less than 200 days were represented by cows who interrupted their lactation period by some process or underwent interference in handling during the period of data collection.

In addition, variable relative contribution for divergence by Singh criteria (1981) was calculated (Table 5). Cluster analysis considers the distances among females for generating groups, and these distances are influenced by traits presenting the highest variability. Thus, the most relevant variables were TMY, with 71.92% of variation, and AFC, with 23.06% of variation, indicating higher selection efficiency for these functional traits. The traits with lower variability and thus considered less important were CI, LP, and RE, with 4.37, 0.61,and 0.02% of the total variance, respectively. These traits have mostly contributed for divergence among cows, being responsible for higher or lower similarity.

According to literature review, age at first calving (AFC) is very important for milk production systems, whereas, as the smaller, as soon the cow become more productive, allowing a greater number of offspring’s during its reproductive life and greater intensity of breeding, which will reflect in higher milk yield. Conversely, when high in general, this reflects the lack of adaptation of animals to their environment (Silva et al. 1998; Pelicioni et al. 1999; Wenceslau et al. 2000; Ettema and Santos 2004; Facó et al. 2005; Coelho et al. 2009; Vieira et al. 2010; Krpálková et al. 2014; Van Pelt et al. 2016; Heise et al. 2018). In our study, the traits total milk yield (TMY) and age at first calving (AFC) contributed the most to cluster of the Red Sindhi dairy cows.

In our study, AFC has highly been correlated with the most relevant components, indicating its relevant contribution for the evaluation of the milk production systems, aiming more sexually precocious females and consequently more productive ones. This fact is due to the fact that the Red Sindhi breed comes from the Northeast region, whereas the selection methods for higher milk yield and herd’s sexual precocity are addressed. On that account, based on the results from our study, dairy cows from groups 7 and 9 (the highest TMY) and those ones from groups 11 and 12 (the lowest AFC) might be employed focusing on milk yield and sexual precocity.

This study investigated the relationships among AFC, CI, RE, LP, and TMY using principal component and clustering analyses using Red Sindhi cows kept under different handling conditions. Although results were based on collected data, these results showed important biological associations underlying the phenotypic relationships for many traits that are important for Red Sindhi dairy cattle breeding programs.

Principal components could be used for all functional traits and would be useful in both dimension reduction and avoiding collinearity problems, common in the analysis of closely related functional traits such as TMY or RE. Clustering analysis also showed the clearly understandable patterns of physiological relationships among functional traits, whereas CI is correlated with RE, and all these functional traits are related with TMY. Since the sampling size was not large, results should still be interpreted carefully and confirmed in other Red Sindhi dairy cattle populations.

## Electronic supplementary material

ESM 1 (DOCX 114 kb)
