# Supplementary material for: Principal component and cluster analyses of production and fertility traits in Red Sindhi dairy cattle breed in Brazil
Source: Trop Anim Health Prod. 2019 Aug 1;52(1):273–81. doi: 10.1007/s11250-019-02009-7 (PMC6969864; doi:10.1007/s11250-019-02009-7)
Supplement: Supplementary file 1 — (DOCX 114 kb) [file 11250_2019_2009_MOESM1_ESM.docx]

Manuscript TROP-D-18-00871: **"Principal component and cluster analyses of production and fertility traits in Red Sindhi dairy cattle breed in Brazil”**

*Supplementary data file:* some information about correct background of the breed with import history and photos, using the references suggested in the comments of the reviewer.

Red Sindhi cattle are the most popular of all [zebu](https://en.wikipedia.org/wiki/Zebu) dairy breeds. The breed originated in the [Sindh](https://en.wikipedia.org/wiki/Sindh) province of [Pakistan](https://en.wikipedia.org/wiki/Pakistan) (Kohistan area), and they are widely kept for milk production across [Pakistan](https://en.wikipedia.org/wiki/Pakistan), [India](https://en.wikipedia.org/wiki/India), [Bangladesh](https://en.wikipedia.org/wiki/Bangladesh), [Sri Lanka](https://en.wikipedia.org/wiki/Sri_Lanka), and other 33 countries, being originated in [Hyderabadand](https://en.wikipedia.org/wiki/Hyderabad,_Sindh) Bikaner. The breed is also known as “Malir”, “Red Karachi” and “Sindhi”, and it is considered that breed is evolved from Las Bela cattle of Bela, Baluchistan. They have been used for crossbreeding with temperate (european) origin dairy breeds in many countries to combine their tropical adaptations (heat tolerance, tick resistance, disease resistance and fertility at higher temperatures) with the higher milk production found in temperate regions. It has been crossed with [Jerseys](https://en.wikipedia.org/wiki/Jersey_cattle) in many places, including India, the United States, Philippines, Australia, Sri Lanka and Brazil.

Red Sindhi range in color from a deep reddish brown to a yellowish red, but most commonly a deep red. They are distinguished from the other dairy breed of [Sindh](https://en.wikipedia.org/wiki/Sindh), the [Tharparkar](https://en.wikipedia.org/wiki/Tharparkar_(cattle)) or White Sindhi, both by color and form, the Red Sindhi is smaller, rounder, with a more typical dairy form, and with short, curved horns, while the Tharparkar are taller with a shape more typical of [zebu](https://en.wikipedia.org/wiki/Zebu) draft breeds, and with longer, lyre shaped horns. Males are darker than females and when mature may be almost black on the extremities, such as the head, feet and tail. There are also many points of resemblance to the Sahiwal cattle, whose origins are also in the red-type mountain cattle of the northern border of India.

Red Sindhi breed originates from a mountainous region called `Mahal Kohistan', spread over parts of Karachi, Thattha and Dadu districts in Sindh. The hometract of this breed extends to the irrigated areas of Hyderabad (Sindh) and the arid plains of Lasbella, district in Balochistan. Due to the territorial extension of the Red Sindhi herd, a variety of strain can be saw outside their original area, and for this reason, when it comes to the choice of pure breeders, there is a tendency to pick them up at Lasbella.

Most breeders are nomads belonging to the Malders tribe, who lead cattle from one place to another in search of good pasture. In the Kohistan area, livestock are exclusively kept under pasture, in natural or barns fields. However, there are many breeders who have settled in Karachi, Hyderabad and other major outlying areas due to the great demand for milk and cows to supply the urban populations. In these places, since forage is scarce, breeders feed with concentrates, especially for lactating cows. These breeders perform milking twice a day, at which time the food is distributed. Although cows are very docile and easy to be milked without breeding, early weaning is not done.

In India, Red Sindhi breed are considered a dairy breed, and males are also used for traction of wagon. Its contingent in Pakistan is approximately four million (population of 4.73 million), which makes it is more established among all of its group, including Sahiwal (population of 4.30 million). In Pakistan, the herd of the Hosur Experimental Farm in the Madras region is older than others, and its selection has been developing satisfactorily. Another well-known herd is the Allahabad Institute of Agriculture. In the northwest of the Pundjab, at the Experimental Farm of Karnal, is a good dairy herd. Red Sindhi breeders have been exported to Ceylon, Philippines, Taiwan, Indochina, Malacca, Burma, Africa, the United States and Jamaica.

Actually, there is a growing number of farms currently exploiting milk in Pakistan using Red Sindhi breed. Since the 1950s crosses involving local breeds have been made with bulls of the Holstein and Jersey breeds improved for milk in order to develop a new productive local type. This attempt has generated positive results, but presents risks for the maintenance of local purity, mainly due to the extensive breeding and nomadic character of the region, and it is also a complication that these crossbreed animals need more and better food quality, often unavailable, which deserves more attention on the part of researchers, authorities and producers.

In 1980s, dairy sector in Pakistan moved towards commercial side and development of rural commercial dairy farms started. A typical rural dairy farm running on commercial basis consisted of about 30 animals of which 70% were females, including some cows. Approximately 40% of these adult females were in milk during most of the year. Fodder crops provided 50% and straws about 35% of the feed requirements and concentrates made the rest of it. More than 90% of the milk produced at the farm was sold.

Red Sindhi has been crossed with [Holstein-Friesian](https://en.wikipedia.org/wiki/Holstein_cattle), [Brown Swiss](https://en.wikipedia.org/wiki/Brown_Swiss) and [Danish Red](https://en.wikipedia.org/wiki/Danish_Red_Cattle). It has also been used to improve beef and dual-purpose cattle in many tropical countries, as it is sufficiently meaty to produce good beef calves in such crosses and the high milk production helps give a fast-growing calf which is ready for market at one year. It is somewhat smaller than the very similar [Sahiwal](https://en.wikipedia.org/wiki/Sahiwal_cattle) and produces a little less milk per animal as a result. This has caused it to lose favor with some commercial dairies in India and Pakistan, which have been phasing out their Red Sindhi herds by breeding to Sahiwal bulls for a few generations. The resulting cows, which are three-quarters Sahiwal and one-quarter Red Sindhi, cannot be distinguished from pure [Sahiwal cattle](https://en.wikipedia.org/wiki/Sahiwal_cattle). Red Sindhi cattle are also used for milk production in [Brazil](https://en.wikipedia.org/wiki/Brazil), but this breed of zebu is not popular as others.

Red Sindhi has medium-sized breed with a compact build and red body color; large head with an occasional bulge in the forehead; horns are thick and stumpy in males but thin in females; ears are fine and small; hump is well‑developed in males; dewlap is moderate in both sexes; sheath is pendulous in males, but nominal in females; hind quarters are round and drooping; tail switch is black; udder is medium to large and strong. Red Sindhi animals are hardy and adapt very well to stressful environments, with the milk yield per lactation varies from 1200 to 2000 litres; fat percentage in the milk varies from 4 to 5.2%, with an average of 4.5%; adult males weigh 400-500 kg, with average height of 134 cm, while females weigh 300-350 kg, with average height of 116 cm; average lactation length is 265 days; age at first calving is 45 months; service period is 210 days; dry period is 230 days; calving interval is 495 days, and gestation period is 288 days.

In conclusion, during last ten years major changes has been occurred in dairy sector of Pakistan. A large number of modern dairy farms established in different areas. Most of these dairy farms have exotic animals and number of these animals is in hundreds and even in thousands. Dairy farms with more than 3000 animals also exist and with 5000 animals are in plan. Such farms have adopted most modern managemental and feeding practices and well-trained man power. Milk produced on these farms is either sold out in processed/fresh form through outlets/ departmental stores/house supply or supplied to dairy companies.

Red Sindhi Photos:

Female adult:


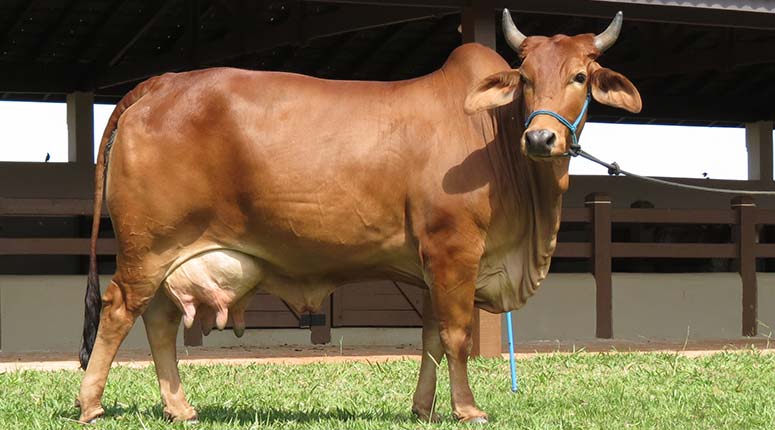


Source: Mello (2014)

Male adult:


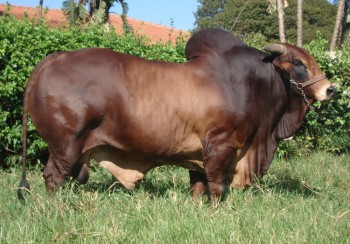


Source: Mello (2014)

*Supplementary Table:* Sindhi females’ groups established by the Tocher optimization method based on the dissimilarity expressed by Euclidean distance standardized mean.

| **Groups** | **N** | **Genotypes** |
| --- | --- | --- |
| **1** | **429** | 192; 219; 127; 104; 248; 520; 375; 556; 297; 447; 356; 426; 263; 165; 281; 392; 85; 195; 68; 131; 325; 108; 203; 386; 29; 76; 488; 46; 417; 241; 27; 419; 500; 407; 299; 83; 402; 384; 266; 43; 313; 184; 258; 89; 399; 398; 415; 223; 435; 528; 405; 519; 368; 53; 45; 61; 344; 421; 514; 332; 225; 48; 521; 308; 558; 97; 5; 358; 416; 262; 410; 216; 450; 240; 93; 204; 44; 413; 425; 71; 269; 472; 550; 323; 26; 73; 47; 264; 191; 56; 41; 13; 198; 510; 1; 151; 156; 362; 403; 333; 270; 509; 138; 49; 218; 179; 327; 486; 96; 538; 69; 328; 324; 227; 113; 177; 169; 307; 228; 230; 409; 217; 122; 211; 166; 207; 463; 551; 492; 285; 142; 188; 314; 348; 279; 160; 161; 91; 350; 55; 451; 489; 147; 390; 522; 128; 255; 178; 329; 15; 21; 267; 482; 537; 560; 273; 157; 173; 287; 51; 95; 237; 493; 317; 24; 88; 494; 548; 342; 474; 549; 397; 460; 257; 146; 99; 535; 8; 132; 484; 322; 78; 65; 495; 557; 58; 172; 52; 243; 134; 394; 414; 433; 539; 239; 33; 40; 170; 312; 148; 374; 411; 140; 382; 159; 364; 175; 197; 289; 220; 505; 102; 143; 502; 432; 559; 36; 154; 190; 387; 251; 369; 278; 542; 155; 212; 280; 276; 352; 523; 130; 293; 541; 512; 331; 144; 457; 401; 428; 391; 527; 265; 291; 371; 14; 126; 162; 50; 64; 292; 487; 490; 116; 209; 90; 544; 330; 176; 4; 193; 353; 452; 114; 545; 215; 242; 149; 181; 393; 38; 458; 183; 86; 98; 552; 79; 72; 275; 420; 139; 288; 277; 82; 12; 57; 101; 310; 200; 479; 359; 395; 206; 274; 199; 504; 443; 205; 141; 226; 137; 381; 444; 301; 30; 124; 210; 185; 87; 378; 442; 305; 158; 18; 424; 477; 286; 19; 336; 187; 63; 284; 385; 174; 16; 318; 208; 453; 372; 339; 110; 247; 326; 54; 163; 246; 136; 233; 306; 167; 20; 478; 28; 112; 271; 202; 250; 303; 180; 260; 357; 229; 213; 476; 125; 346; 354; 383; 355; 32; 311; 66; 473; 316; 349; 449; 309; 282; 319; 376; 431; 70; 379; 37; 145; 412; 182; 189; 534; 302; 321; 283; 171; 290; 152; 365; 92; 434; 168; 377; 462; 84; 261; 164; 465; 335; 23; 461; 497; 367; 360; 253; 135; 34; 35; 511; 438; 427; 244; 506; 7; 471; 234; 531; 400; 389; 39; 468; 17; 501; 526; 106; 513; 150; 60; 373; 525; 543; 11; 343 |
| **2** | **63** | 214; 464; 232; 296; 341; 515; 408; 483; 337; 340; 119; 245; 508; 222; 499; 10; 436; 469; 533; 554; 236; 546; 118; 524; 454; 186; 459; 62; 6; 67; 532; 530; 440; 380; 441; 129; 351; 315; 437; 422; 224; 555; 366; 107; 467; 363; 455; 294; 529; 334; 370; 74; 536; 361; 75; 300; 121; 59; 540; 31; 201; 105; 466 |
| **3** | **23** | 338; 406; 498; 249; 516; 429; 298; 231; 439; 418; 235; 272; 496; 153; 109; 259; 238; 518; 268; 133; 252; 345; 117 |
| **4** | **15** | 485; 491; 553; 388; 103; 475; 503; 81; 196; 111; 396; 123; 470; 446; 94 |
| **5** | **8** | 25; 194; 22; 9; 347; 221; 254; 320 |
| **6** | **13** | 404; 448; 256; 100; 480; 507; 115; 547; 120; 423; 304; 481; 517 |
| **7** | **2** | 445; 456 |
| **8** | **2** | 3; 77 |
| **9** | **2** | 295; 430 |
| **10** | **1** | 42 |
| **11** | **1** | 80 |
| **12** | **1** | 2 |

N = number of cows in each group.

References:

<https://en.wikipedia.org/wiki/Red_Sindhi>

[http://www. pakdairyinfo.com/redSindhi.htm](http://www.pakdairyinfo.com/redSindhi.htm)

<http://dairyknowledge.in/article/red-sindhi>

<http://14.139.252.116/agris/bridDescription.aspx>

<http://afs.okstate.edu/breeds/cattle/redsindhi/index.html/>

<http://www.sindi.org.br/noticia.php?id=69>

<http://www.worldcat.org/title/red-sindhi-cattle/oclc/13224514>

own sources: Mello (2014)
